# Cascaded Effects of Spatial Adaptation in the Early Visual System

**DOI:** 10.1016/j.neuron.2013.11.025

**Published:** 2014-02-05

**Authors:** Neel T. Dhruv, Matteo Carandini

**Affiliations:** 1 UCL Institute of Ophthalmology, University College London, 11-43 Bath Street, London EC1V 9EL, UK

## Abstract

Virtually all stages of the visual system exhibit adaptation: neurons adjust their responses based on the recent stimulus history. While some of these adjustments occur at specific stages, others may be inherited from earlier stages. How do adaptation effects cascade along the visual system? We measured spatially selective adaptation at two successive stages in the mouse visual system: visual thalamus (LGN) and primary visual cortex (V1). This form of adaptation affected both stages but in drastically different ways: in LGN it only changed response gain, while in V1 it also shifted spatial tuning away from the adaptor. These effects, however, are reconciled by a simple model whereby V1 neurons summate LGN inputs with a fixed, unadaptable weighting profile. These results indicate that adaptation effects cascade through the visual system, that this cascading can shape selectivity, and that the rules of integration from one stage to the next are not themselves adaptable.

## Highlights

• Different stages of the visual system show different forms of spatial adaptation
• In LGN, adaptation controls only response gain; in V1, it also controls tuning
• These disparate effects are reconciled by a summation model with fixed weights
• The cascade of adaptation shapes tuning without affecting the summation rules

## Teaser

How does sensory adaptation in one brain region affect downstream regions? Here, Dhruv and Carandini show how a simple form of subcortical adaptation cascades onto visual cortex, affecting not only the responsiveness but also the selectivity of downstream neurons.

## Introduction

Since the very first report of spike trains in sensory nerves (Adrian and Zotterman, 1926), there have been multiple demonstrations of neural adaptation in sensory systems. Through adaptation, sensory systems adjust their activity based on recent stimulus statistics (Wark et al., 2007). These effects are pervasive: they are observed in invertebrates (Brenner et al., 2000; Fairhall et al., 2001) and in vertebrates, where they affect multiple sensory modalities, including somatosensation (Maravall et al., 2007), audition (Condon and Weinberger, 1991; Dean et al., 2005; Nagel and Doupe, 2006; Ulanovsky et al., 2003), and vision (reviewed in Kohn, 2007).

In the visual system, in particular, adaptation appears to operate at all stages, including retina (Smirnakis et al., 1997), lateral geniculate nucleus (LGN; Solomon et al., 2004), primary visual cortex (V1; reviewed in Carandini, 2000; Kohn, 2007), and primate cortical area MT (Kohn and Movshon, 2003, 2004). In V1, for instance, adaptation has two main effects (Benucci et al., 2013; Kohn, 2007): it controls neuronal responsiveness based on the strength of recent stimulation (Carandini and Ferster, 1997; Ohzawa et al., 1982; Sanchez-Vives et al., 2000), and it shifts neuronal selectivity away from recently viewed stimuli (Dragoi et al., 2002; Movshon and Lennie, 1979; Müller et al., 1999). The first effect is akin to general neural fatigue; the second suggests a more specific adjustment of stimulus representation.

There is little doubt that neural adaptation is intimately related to, and must ultimately explain, the long-known phenomena of perceptual adaptation. However, neural adaptation has been overwhelmingly studied in neurons of individual brain regions. To establish its origins and predict its overall effects, we need to understand how it cascades across brain regions.

While some adaptation effects originate in the area where they are observed, others may be inherited from earlier stages. For instance, many of the adaptive changes observed in the LGN are probably inherited from retina (Solomon et al., 2004). Similarly, some effects of adaptation observed in V1 may stem from changes in the geniculate input (Dhruv et al., 2011). Finally, part of the adaptation effects observed in primate MT could be inherited from V1 (Kohn and Movshon, 2003, 2004).

If we know how adaptation affects one brain region, can we predict how it affects a second, downstream brain region? The second region will inherit adaptation from the incoming spike trains. In addition, adaptation may affect the way the second region integrates those spike trains. For instance, it could change the strength of incoming synapses.

To investigate how adaptation effects cascade through the visual system, we focused on the geniculocortical pathway, which has long served as a test bench to characterize how signals are affected by integration from one region to the next. The rules by which V1 integrates LGN inputs are well understood (Alonso et al., 2001; Kara et al., 2002), but it is not known whether these rules are themselves adaptable. We found that spatial adaptation affected responses in both LGN and V1, but it did so in profoundly different manners. We could reconcile these differences by implementing an extremely simple integration model that is not itself modified by adaptation.

## Results

**Figure 1:**
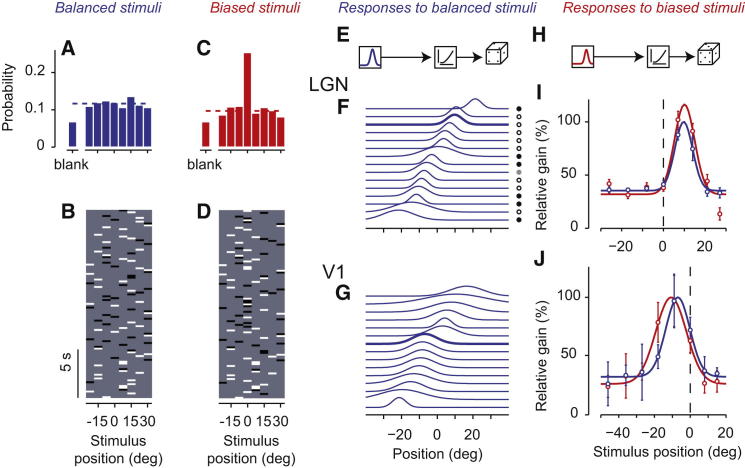
Stimuli and Examples of Results (A and B) Balanced stimulus. (C and D) Biased stimulus. Stimulus examples (B and D) are 20 s samples from the full stimulus sequence (typically ∼10 min). Stimulus histograms (A and C) are computed from the full stimuli. (E) Linear-Nonlinear-Poisson (LNP) model used to describe responses to balanced stimuli. (F) Examples of tuning curves obtained for 15 neurons in LGN. Position is expressed relative to the position that will be used as adaptor in the biased condition. (G) Same, for 15 neurons in V1. Open and closed circles denote ON and OFF center cells. (H) LNP model for responses to biased stimuli. (I) The tuning curve of an LGN neuron (thick curve in F) measured in response to balanced stimuli (blue) and biased stimuli (red). The gain, or responsiveness, at each position was normalized to the peak value measured in the balanced condition. Curves are best-fitting Gaussians. Error bars indicate two SDs in the estimate. (J) Same, for a V1 neuron. See also Figure S1.

To measure adaptation, we mapped receptive fields in LGN and V1 with noise sequences whose statistics were either balanced or biased (Figures 1A–1D). This approach allows one to simultaneously induce and probe the effects of adaptation (Baccus and Meister, 2002; Benucci et al., 2013; Brenner et al., 2000; Fairhall et al., 2001; Smirnakis et al., 1997). We presented vertical bars at six to nine locations in random order and with random polarity (white or black). In balanced sequences, the probability of presenting a stimulus at any position was equal (Figures 1A and 1B). In biased sequences, instead, a given position, the adaptor, was two to three times more likely than the other positions (Figures 1C and 1D).

We first used the balanced stimuli and characterized the receptive field profiles (Figures 1E–1G). We fitted the neural responses with a Linear-Nonlinear-Poisson (LNP) model (Figure 1E), which is a well-established functional characterization (Paninski, 2004; Pillow, 2007; Simoncelli et al., 2004). The model provided an accurate description of the responses, as judged, for instance, by its ability to replicate the average stimulus-triggered responses (Figure S1 available online). The linear stage of the model is a filter in space and time, which operates on signed contrast (for well-isolated LGN neurons and V1 simple cells) or on unsigned contrast (for MUA and for V1 complex cells). The spatial aspect of this filter constitutes an envelope of the neuron’s receptive field profile, which was typically well fitted by a Gaussian curve (Figure S1). As expected, receptive field profiles were considerably narrower in LGN than in V1 (e.g., Figures 1F and 1G), with a half-width of 5.3° ± 1.9° in LGN (n = 86) versus 10.5° ± 4.8° in V1 (n = 29). These measurements are in line with previous estimates both for LGN (6°; Grubb and Thompson, 2003) and for V1 (7°–15°; Niell and Stryker, 2008; Van den Bergh et al., 2010).

We then asked whether and how these receptive field profiles adjust to biases in the stimulus statistics (Figures 1H–1J). We fitted the LNP model to the responses to the biased stimuli, forcing the nonlinearity to be the same for balanced and biased stimuli. The effects of adaptation were captured, therefore, by changes in the receptive field profile (Figures 1I and 1J). The value of this profile at each position is a measure of responsiveness, or gain, at that position, and we expressed it relative to the value measured at the best position in the balanced condition.

We saw two types of changes. In some cases, the receptive field profile only changed in amplitude, i.e., in responsiveness (e.g., Figure 1I). In other cases, there was a clear shift in preferred position, corresponding to a change in tuning (e.g., Figure 1J). As we will see, the first effect was reliably seen in LGN and the second was consistently observed only in V1.

**Figure 2:**
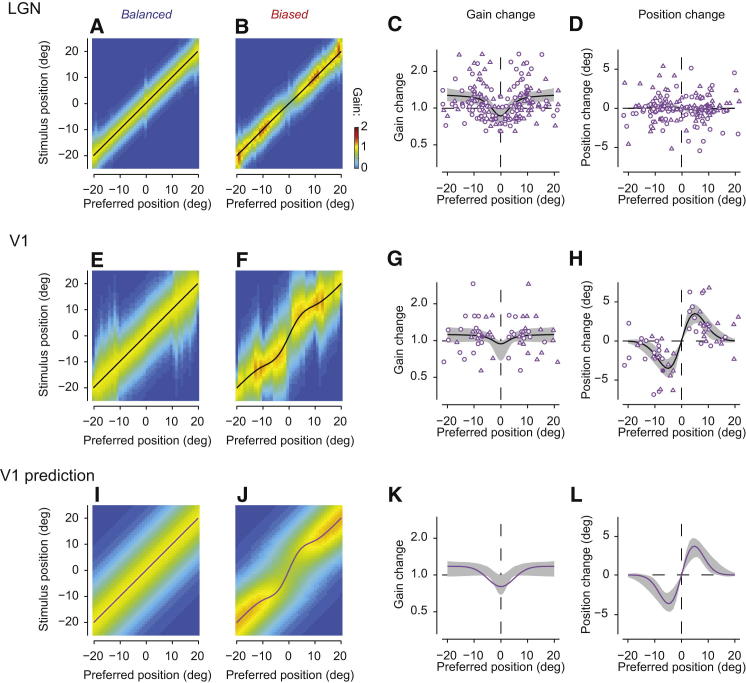
Effects of Adaptation on LGN and V1 Tuning Curves and Population Responses (A and B) Full response matrix of LGN in balanced (A) and biased (B) conditions, computed from sliding window bin of fixed width (4°) across preferred position. For clarity, we have symmetrized the data by averaging data with the same absolute distance from the adaptor position. Black lines trace the preferred stimulus for each neural bin. (C and D) Difference in gain and preferred position of LGN receptive field profiles computed for biased and balanced conditions. Thick black lines and gray fields indicate median and 90% confidence intervals of bootstrap fits to the data. Curves fitted to gain changes (C) are Gaussians, and curves fitted to position changes (D) are Gabor functions. Measured points are indicated by circles. Triangles indicate their mirror-symmetric duplicates. (E–H) Same conventions as (A)–(D) for corresponding measurements in V1. Filled symbols in (C), (D), (G), and (H) refer to example cells in Figures 1I and 1J. n = 86 for (A)–(D) and n = 29 for (E)–(H). (I–L) Same conventions as (A)–(D) for the predictions of the fixed summation model of V1 responses (described in Figure 3). See also Figure S2.

In LGN neurons, the main effect of adaptation was to scale the response gain, without changing the receptive field profile (Figures 2A–2D). We summarize the effects of adaptation on the LGN population by plotting responsiveness as a function of stimulus position and of each neuron’s preferred position (Figures 2A and 2B). To obtain this plot, we normalized each cell’s tuning curve to that determined in the balanced condition, we pooled cells whose preferred position fell within a 4° bin, and we computed the median response in each bin. As expected, for balanced sequences the resulting plot is diagonal, since a neuron’s preferred position is defined by the stimuli that evoke the largest response (Figure 2A). For biased sequences, instead, there was an increase in response gain for neurons having preferred position distant from the adaptor, which is given the nominal position of zero (Figure 2B). In addition, there was a decrease in gain for neurons whose receptive field substantially overlapped with the adaptor.

These effects are most clearly seen by plotting response gain as a function of preferred position relative to the adaptor (Figure 2C). The LGN neurons that responded to the adaptor were desensitized by the increase in stimulus frequency. The remaining neurons instead showed the opposite effect, perhaps due to the decreased frequency of the remaining stimuli or to adaptation of their nonclassical suppressive field (see Discussion). Most importantly, however, these gain changes appeared without a systematic change in the preferred tuning of a cell: on average, the neurons preferred the same position in the two adaptation conditions (Figure 2D).

The effects of adaptation in V1 neurons were manifestly different: receptive field profiles showed a marked repulsion (Figures 2E–2H). This repulsion distorted the relationship between stimulus position and preferred position (Figure 2F). The maximum repulsion occurred for V1 cells with receptive field profiles peaking ∼5° away from the adaptor (Figure 2H). The receptive field profiles of these cells were shifted by ∼3.5°. Given the typical tuning width (full-width at half-height [FWHH]) of 21°, this equates to a shift of ∼17%. These marked shifts in preference were accompanied by small changes in response gain (Figure 2G) and minor changes in tuning width (data not shown). These effects did not seem to depend on cortical layer and appeared to be weaker in some putative inhibitory interneurons, as judged by spike width (Figure S2).

How can the same kind of adaptation regime impact two adjacent stages of processing so differently? One possibility is that adaptation changes the way that V1 operates on signals from the LGN. In particular, perhaps it changes the way that V1 neurons summate their LGN inputs, enhancing the contribution of LGN neurons tuned for positions that are distant from the adaptor. Alternatively, V1 might be unaware of spatial adaptation and inherit it entirely from the changes that adaptation causes in LGN. Indeed, even if the summation rules between LGN and V1 remained fixed, V1 neurons would integrate over different profiles of LGN activity depending on the adaptation condition. If this “cascade hypothesis” could account for the data, it would be preferable for its parsimony.

The cascade hypothesis was indeed sufficient to account for the data (Figures 2I–2L). We considered a fixed summation model where V1 neurons obtain their spatial selectivity through a weighted sum of the appropriate LGN inputs, with weights that are not adaptable. We then applied this model to LGN responses determined from our measurements (Figure 2I). The predicted V1 responses (Figure 2J) closely resembled the measured ones (Figure 2F): they showed a mild reduction in gain at the adaptor position (Figure 2K) and a clear repulsion of the tuning curves away from that position (Figure 2L). Overall, the model accounted for ∼98% of the variance in the V1 responses, and the residuals (data not shown) did not show much structure. The fixed summation model, therefore, provides a good account of the effects of spatial adaptation in V1.

**Figure 3:**
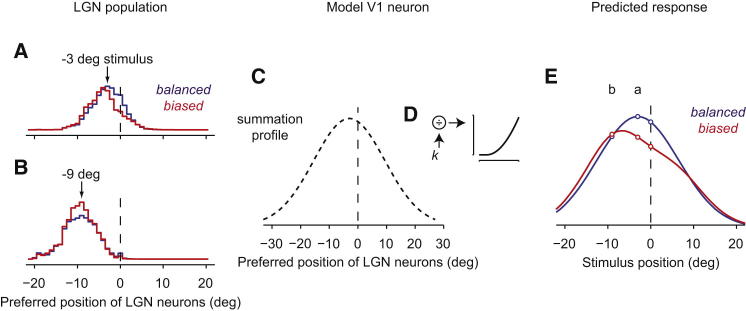
The Fixed Summation Model of V1 Responses (A) LGN population response evoked by a stimulus presented at −3° in the balanced (blue) or biased (red) conditions. Vertical line indicates position of the adaptor. (B) Same, for a stimulus presented at −9°. (C) Summation profile of a V1 cell with a receptive field centered on LGN neurons tuned for −3°. (D) Postsummation nonlinearity and gain control. (E) The model V1 cell prefers −3° in the balanced condition (blue) and −6.5° in the biased condition (red). See also Figure S3.

To illustrate the workings of the model, consider its predictions for the responses of a V1 neuron to two stimuli (Figure 3). Take first a stimulus that is close to the adaptor, 3° away. This stimulus elicits a profile of LGN activity that is barely affected by adaptation (Figure 3A). Next, take a stimulus that is further away from the adaptor, 9° away. This stimulus elicits a profile of LGN activity that is strongly enhanced by adaptation (Figure 3B). Now consider a V1 neuron that summates LGN inputs with weights that peak for LGN neurons preferring −3° (Figure 3C). As is typical for V1 neurons, the output of this sum is then passed through a stage of divisive normalization (Carandini and Heeger, 2012) and a static nonlinearity (Priebe and Ferster, 2008), neither of which depends on spatial position (Figure 3D). This model V1 neuron exhibits rather different tuning curves depending on the adaptation condition (Figure 3E). In response to balanced sequences, the tuning curve is centered on −3° and therefore resembles the weighting function (Figure 3E, blue). In response to biased sequences, instead, the tuning curve is shifted away (Figure 3E, red).

This example illustrates how the tuning curves of model V1 neurons are repelled by the adaptor even though adaptation does not affect the summation weights. Normalization and the static nonlinearity play no role and are present in the model simply to explain response amplitudes. Normalization, in particular, divides the output of all V1 neurons to all stimuli in the sequence by a common factor *k* (Figure 3D). This factor happens to be somewhat larger in the biased condition (Figure S3), but it cannot change the resulting tuning curves. Rather, the tuning curves of model V1 neurons are repelled because their inputs from remote LGN neurons are disproportionately enhanced.

**Figure 4:**
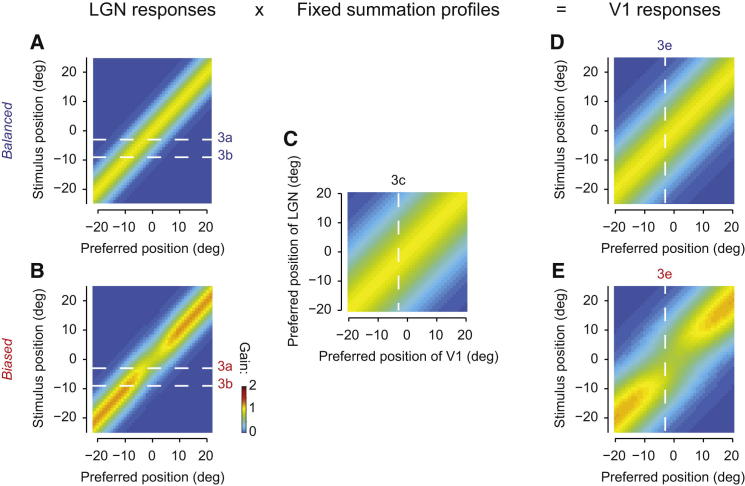
The Fixed Summation Model Expressed as Matrix Operations (A and B) Idealized LGN population response in the balanced (A) or biased (B) conditions. Response matrices are constructed from the Gaussian fit in Figure 2C and the Gabor fit in Figure 2D. Horizontal dashed lines correspond to example stimuli in Figures 3A and 3B. (C) Best-fitting V1 summation profile expressed as a function of LGN and V1. Dashed line represents example summation profile in Figure 3C. (D and E) Idealized V1 population response in the balanced (D) and biased (E) conditions. Response matrices are constructed from fitting the fixed summation model to the V1 balanced and biased data given the input LGN response matrices. Dashed lines represent example V1 cell in Figure 3E. Panels (D) and (E) have been replicated as Figures 2I and 2J for the sake of comparison. Static nonlinearity and gain control factor as described in Figure 3 are not shown in this figure but were part of the fit.

To understand this summation model further, it helps to cast it in terms of matrix operations (Figure 4). The model operates on matrices of LGN responses expressed as a function of neuronal preference and of stimulus position. In the balanced condition, this response matrix is simply diagonal (Figure 4A): the responses of each LGN neuron depend only on the distance between stimulus position and preferred position. We obtain this response matrix by assuming that LGN neurons tile visual space and have identical tuning width (FWHH ∼10.6°, the median value in our population). In the biased condition, we modify this response matrix by changing the gain of the LGN neurons depending on their preferred position relative to the adaptor (Figure 4B). We obtain the new gain values from the fit to the LGN data (Figure 2C). The responses of model V1 neurons are then obtained by multiplying the matrix of LGN responsiveness by a matrix of summation weights, which describe the tuning of V1 neurons over their geniculate inputs. Extended to the full V1 population, the summation profile becomes a diagonal matrix, whose values depend on the strength and breadth of the convergence from LGN to V1. We assume that this matrix is not affected by adaptation (Figure 4C).

Once we found the optimal parameters of the summation profile, we used them to predict the matrices of responsiveness observed in V1 (Figures 4D and 4E). The best-fitting exponential was ∼1.7, and the width of the summation Gaussian (FWHH) was ∼28° (Figures 3C and 4C). In the unbiased condition, the model correctly predicted the diagonal structure of the V1 matrix (Figure 4D). In the biased condition, more importantly, the model fitted both the repulsion of tuning curves and the shape of the gain change that we observed in V1 (Figure 4E). As we have seen (Figures 2K and 2L), these predictions are accurate even though no model parameters were allowed to vary across adaptation conditions. We could therefore replicate the strikingly different effects of adaptation in LGN and V1 by assuming that V1 is completely blind to spatial adaptation and inherits its effects entirely from the population responses of LGN.

## Discussion

Our results illustrate how adaptation can cause changes that are straightforward in one brain region and then cascade onto the next brain region to produce changes that are more complex and profound. Specifically, we found that spatial adaptation has markedly different effects in LGN and V1: in LGN, it only changes response gain, but in V1, it also changes stimulus selectivity. We explained these disparate effects by using a summation model with fixed weights. According to this model, spatial adaptation cascades onto V1, shaping the tuning of its neurons without affecting their summation of LGN inputs.

Our results are in general agreement with previous studies of cascading adaptation measured physiologically (Kohn and Movshon, 2003, 2004). These studies compared adaptation to motion in primate areas V1 and MT and found that it changed the tuning curves in area MT but not in area V1. The authors suggested that a cascade model similar to ours could account for the observed effects, i.e., that MT neurons could inherit their adaptation properties from adaptation in their inputs. More recent work indicates that adaptation can change fundamental attributes of how MT neurons integrate motion patterns, and yet that these changes can be entirely inherited from gain changes occurring in area V1 (Patterson et al., 2014). In fact, the model we used for how V1 neurons process LGN inputs resembles a widely accepted model for how MT neurons process V1 inputs: a weighted sum followed by a normalization stage and a static nonlinearity (Rust et al., 2006).

However, our results do not mean that each stage of the visual system merely inherits adaptation from its inputs. Different stages can add adaptation to specific features to which they are sensitive. For instance, since LGN neurons of cats and primates are not selective for stimulus orientation, they could not be responsible for the powerful effects of adaptation seen in V1 in the orientation domain (Benucci et al., 2013; Kohn, 2007).

These results will help interpret the effects of neural adaptation that are routinely measured in electrophysiology and in a multitude of fMRI measurements. In fMRI studies, neural adaptation is often used to estimate the sensory properties of a given brain region and to infer neural selectivity (Krekelberg et al., 2006). However, it is difficult to distinguish effects of adaptation that are inherited from earlier stages from those that are specific to a cortical area, and in some cases adaptation appears to proceed unchanged from one cortical area to the next (Gardner et al., 2005). In the visual system, a promising method to overcome this difficulty is to measure the spatial selectivity of adaptation, exploiting the fact that earlier stages have smaller receptive fields than later stages (S. Harrison and J.Y. Larsson, 2012, Soc. Neurosci., abstract).

In the view of adaptation that emerges from these studies, each stage inherits passively the adaptation provided by the previous stages, without modifying its input rules to help this adaptation or to counteract it. Each stage can then add its own form of adaptation. The goals of this adaptation may differ in different brain regions. For instance, in V1 the goal could be to maintain homeostatic balance across groups of neurons (Benucci et al., 2013).

A similar view has emerged from psychophysical measurements of adaptation. In particular, there is evidence that perceptual effects of motion adaptation on perceived velocity arises from a cascade of two mechanisms, one that knows about visual motion and one that does not (Stocker and Simoncelli, 2009). More generally, our view agrees with the general idea that perception arises from an encoder-decoder cascade, in which the decoder is not aware of the adaptation that occurred in the encoder (Seriès et al., 2009).

Our results identify in the LGN responses the cause for the changes in V1 spatial tuning, but they do not reveal the mechanisms underlying the changes seen in LGN. LGN neurons with receptive fields near the adapting stimulus were reduced in gain relative to the rest. This effect could be inherited from retina or be strengthened in LGN, as both regions show evidence for spatial adaptation (Solomon et al., 2004). However, LGN neurons with receptive fields further away saw an increase in gain. This increase may be due to the slight decrease in probability of stimulation that these neurons experienced in the biased stimuli, or it may be due to adaptation desensitizing their nonclassical suppressive field (Bonin et al., 2005; Camp et al., 2009; Solomon et al., 2002).

Adaptation can radically transform the neural signal as it cascades through the neural hierarchy. We expect this effect to appear wherever the tuning curves of one area build on the population responses of its feedforward inputs. For instance, we would expect similar effects in other sensory domains such as audition. Here, adaptation to a particular sound frequency might scale response magnitude subcortically but shift tuning curves in subsequent stages. The results obtained here, therefore, may apply to multiple brain regions and modalities.

## Experimental Procedures

All experimental procedures were conducted according to the UK Animals Scientific Procedures Act (1986). Experiments were performed at University College London under personal and project licenses released by the Home Office following appropriate ethics review.

### Animals

We recorded from LGN in four anesthetized mice and from V1 in four anesthetized and two awake mice. All but one mouse were C57BL/6, and the remaining one expressed Channelrhodopsin-2 in all layers of cortex under the Thy 1 promoter (Arenkiel et al., 2007). The results can be cumulated because we did not stimulate it optogenetically. Mice were 6–20 weeks old at the time of recording.

### Initial Surgery

We performed surgery under isoflurane gas anesthesia, supplementing it in some animals, with a mixture of ketamine (85 mg/kg, intraperitoneally [i.p.]) and xylazine (7 mg/kg, i.p.). We injected a sedative (chlorprothixene; 10^−5^ mg/kg i.p.), a pain killer (rymadil; 4 mg/kg, subcutaneously), and an anti-inflammatory steroid (colvasone; 2 mg/kg, intramuscularly). We removed the fur and skin over the skull and cleaned the skull before implanting a metal head post. We then made a craniotomy over either LGN or V1, through which we could insert electrodes.

### Acute Experiments

In eight out of ten mice, we measured LGN or V1 responses under anesthesia. After surgery, we administered urethane (1 g/kg, i.p.) and then waited at least 30 min before recording. We monitored the respiration rate, heart rate, and core body temperature throughout the initial surgery and experiment and took appropriate action when needed.

### Chronic Experiments

In two out of ten mice, we measured V1 responses in wakefulness. In these mice, the initial surgery included the implant of a chamber on the skull over visual cortex. The mice recovered for at least 4 days before performing any recordings. We protected the brain in between recording days by filling the chamber with a silicone plug. At the end of the final recording session, we sacrificed the mice with a barbiturate overdose (sodium pentothal; 200 mg/kg, i.p.).

### Recording

We recorded with multisite silicon linear probes (NeuroNexus A1x16; 50 μm spacing, 703 μm^2^ area). We acquired the data at 30 kHz and recovered the activity of single neurons offline with a spike-sorting algorithm (KlustaKwik; Harris et al., 2000). Neurons were included in the study only if their spikes could be isolated from the rest with reasonable accuracy, with median spike isolation distances of ∼17.5 in LGN and ∼24.4 in V1 (Harris et al., 2001; Schmitzer-Torbert et al., 2005) and if they exhibited well-localized receptive fields. We inserted electrodes at coordinates 1 mm anterior and 2.5 mm lateral of lambda for recordings in V1 (Atallah et al., 2012) and 2.5 mm posterior and 2 mm lateral of bregma for recordings in LGN (Grubb and Thompson, 2003). About half of the LGN neurons had receptive fields that were located near the vertical meridian (10°–20° azimuth), while the rest were centered 30°–60° away.

### Stimuli

We presented stimuli using PsychToolbox (Brainard, 1997; Pelli, 1997) on two calibrated LCD monitors (HannsG HW191, mean luminance ∼50 cd/m^2^ or NEC MultiSync, mean luminance ∼40 cd/m^2^) with a frame refresh of 60 Hz. We mapped receptive fields in the horizontal dimension by presenting sequences of vertical bars (∼10° wide) having random position (six to nine positions, spanning 56°–77° in azimuth) and polarity (black or white; Figure 1B). A fraction of the bars (usually 8%) were set to zero contrast to obtain blanks (Figure 1A). Each sequence lasted 20 s, and each bar was flashed for 166 or 200 ms. We generated six such sequences and repeated each five times.

We used two types of random sequences: balanced and biased. In balanced sequences, the bars were equally likely to appear at any position (Figures 1A and 1B). In biased sequences, the bars were two to three times more likely to appear at a given position than at any of the other positions (Figures 1C and 1D). The number of blanks was kept the same.

### Data Analysis

We fit each cell with a Linear-Nonlinear-Poisson model (LNP model) that maximized the likelihood of the observed spike trains (Paninski, 2004; Pillow, 2007; Simoncelli et al., 2004). The nonlinearity was imposed to be the same in the balanced and the biased conditions. In this way, differences in tuning and responsiveness between the balanced and biased conditions are entirely captured by the linear filters. We included a constant offset term so that we could allow for changes in mean activity between the two conditions (Figures 1E and 1H). We fitted two versions of the LNP model for each cell: one in which the linear filter was convolved with a signed version of the stimulus (as appropriate for linear cells), and one in which it was convolved with an unsigned version of the stimulus (as appropriate for nonlinear cells). For each cell, we chose the version of the model that gave the highest likelihood of the data. We selected the time slice at which the linear filters were maximal to obtain the spatial tuning curve of each neuron (Figure S1). We fitted these responses with Gaussian functions (Figures 1F, 1G, 1I, and 1J) and used the appropriate parameters to quantify response gain, preferred position, and tuning width for each neuron.

### Fixed Summation Model

We describe the tuning curve of an LGN neuron as:

(Equation 1) $$R_{LGN}\left(\varphi ,\;\theta_{LGN}\right)=f\left(\varphi -\theta_{LGN}\right)$$

where φ is the stimulus position and f() is the receptive field profile of an LGN neuron with preferred position $\theta_{LGN}$. We can then construct the response of a V1 neuron with preferred position $\theta_{V1}$ to the same stimulus as:

(Equation 2) $$R_{V1}\left(\varphi ,\;\theta_{V1}\right)={\left(\sum_{\theta_{LGN}}R_{LGN}\left(\varphi ,\;\theta_{LGN}\right)g\left(\theta_{LGN}-\theta_{V1}\right)\right)}^{\alpha }$$

where g() is the summation profile of the V1 neuron over LGN. This quantity is integrated over all LGN neurons and passed through a static nonlinearity (α). Effectively, the V1 neuron weights the population response of LGN by its summation profile.

To account for our data, it was sufficient to use simple Gaussian functions to describe both f() and g(). Had we used smaller stimuli and had we tailored their orientation to the preference of V1 neurons, we would have probably needed more complex functions, such as a difference-of-Gaussians for LGN neurons or a modified Gabor function for V1 simple cells (Hawken and Parker, 1987).

### Calculation of Normalization Factor

We computed the normalization factor in each condition by considering the average response in V1 to the balanced and biased stimulus sequences. We first apply the summation profile to the LGN input population to determine the V1 population response prior to normalization. We then compute the normalization factor as:

(Equation 3) $$k=\sum_{s}\left(\sigma^{n}+\sum_{i}L_{is}^{n}\right)p\left(s\right)$$

where $L_{is}$ is the prenormalization response of neuron *i* to stimulus *s*, and p(s) is the probability of stimulus *s.* The constants *σ* and *n* are not allowed to vary between the balanced and biased conditions.
