# Supplementary material for: Cascaded Effects of Spatial Adaptation in the Early Visual System
Source: Neuron. 2014 Feb 5;81(3):529–35. doi: 10.1016/j.neuron.2013.11.025 (PMC3969249; doi:10.1016/j.neuron.2013.11.025)
Supplement: Document S1. Figures S1–S3 [file mmc1.pdf]

Neuron, Volume *81*

Supplemental Information

Cascaded Effects of Spatial Adaptation in the Early Visual System

Neel T. Dhruv and Matteo Carandini

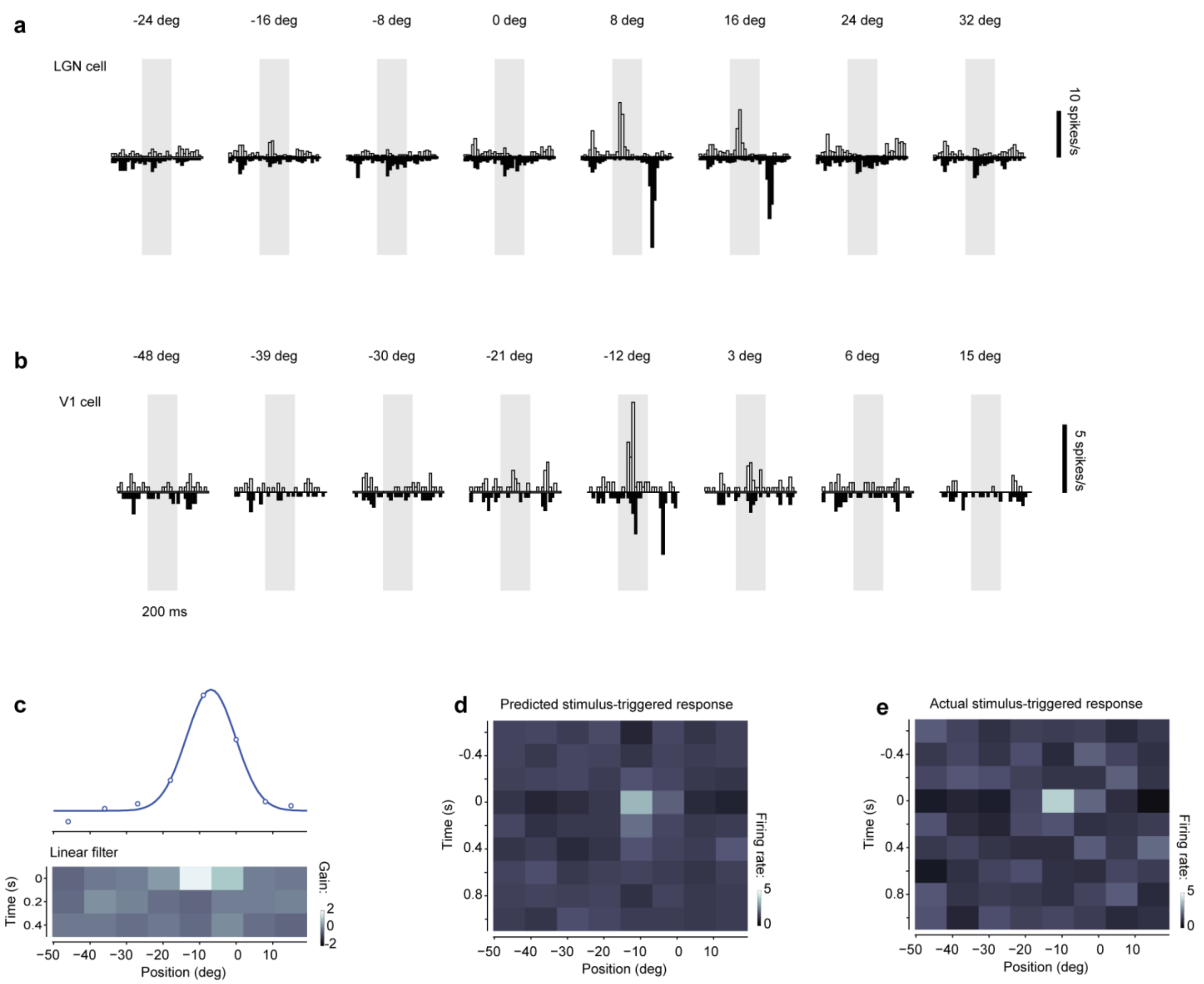

Figure S1: Example cell responses and properties of the linear filter in the LNP model used in Figure 1. **a**: Peristimulus-time histograms of the responses of the LGN cell in Fig. 1**i** (an ON-center cell) to stimuli in the balanced condition, as a function of stimulus position (columns). Upward bars show responses to white bars, downward bars to black bars. **b**: Same, for the V1 cell in Fig. 1**j**. **c**: Linear filter for the same V1 cell. The filter has the dimensions of space and time. The curve is a Gaussian function that best fitted its spatial profile (obtained at the time of peak responsiveness). **d**: Stimulus-triggered responses predicted by the LNP model based on the filter in **c**. **e**: The measured stimulus-triggered responses resemble those predicted by the model, shown in **d**.

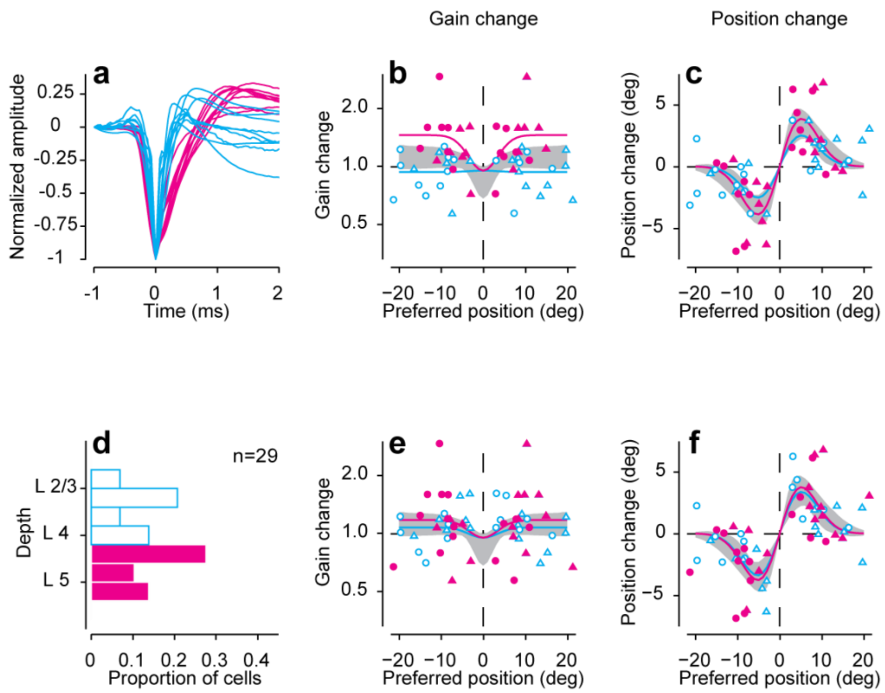

Figure S2: Dependence of gain change and position change on cell-type and cortical depth, for the data shown in Figure 2. **a**: Normalized spike shapes of V1 cells allowing for separation of thin spikes and thick spikes (Bartho et al., 2004). In mouse, the former are thought to be parvalbumin-positive inhibitory interneurons (DeFelipe et al., 2013). **b**,**c**: Same conventions as Figure 2 **g**,**h** but color coded to indicate putative cell type. Solid lines are refits of the descriptive functions to the data subsets indicated. **d** distribution of cortical depth for our population. **e**,**f**: Same conventions as Figure 2 **g**,**h** but color coded to indicate depth division. Solid lines are refits of the descriptive functions to the data subsets indicated.

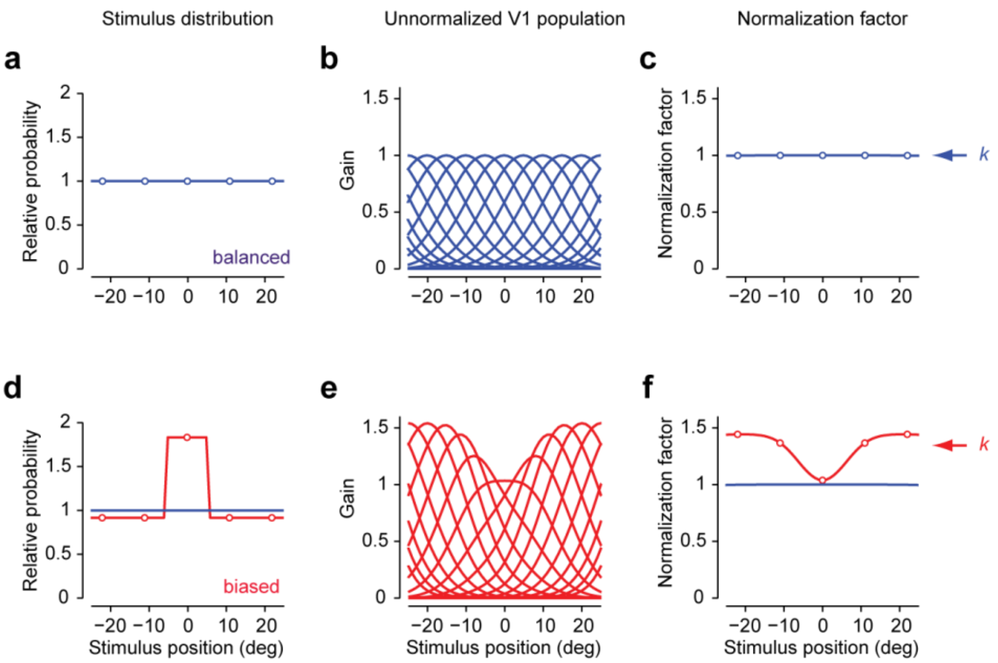

Figure S3: Calculation of normalization factor used in Figure 3. **a**: distribution of relative stimulus probability in the balanced condition. **b**: V1 population prior to normalization in the balanced condition. **c**: Normalization factor as a function of the stimulus in the balanced condition. Arrow indicates average normalization factor. **d**-**f**: Same conventions as **a**-**c** but for the biased condition.

Supplemental References

Bartho, P., Hirase, H., Monconduit, L., Zugaro, M., Harris, K.D., and Buzsaki, G. (2004). Characterization of neocortical principal cells and interneurons by network interactions and extracellular features. J Neurophysiol 92, 600-608.

DeFelipe, J., Lopez-Cruz, P.L., Benavides-Piccione, R., Bielza, C., Larranaga, P., Anderson, S., Burkhalter, A., Cauli, B., Fairen, A., Feldmeyer, D., *et al.* (2013). New insights into the classification and nomenclature of cortical GABAergic interneurons. Nat Rev Neurosci 14, 202-216.
